# A functional framework for a comprehensive study of welfare in fishes

**DOI:** 10.1098/rspb.2025.1833

**Published:** 2025-10-08

**Authors:** Maria Victoria Alvarado, Jose Miguel Cerdá-Reverter, Felipe Espigares

**Affiliations:** ^1^ Fish NeuroBehaviour Laboratory, Department of Fish Physiology and Biotechnology, Instituto de Acuicultura de Torre de la Sal, Castellon, Comunidad Valenciana, Spain

**Keywords:** animal welfare, fish, affective states, judgement bias, behavioural indicators of welfare, physiological indicators of welfare, physical indicators of welfare

## Abstract

Fish production is expected to grow significantly in the coming years. The welfare of captive animals in increasingly intensive systems has historically raised ethical concerns, suggesting that such intensification in fish production will drive public debate and inform policy discussions. Traditional assessments in fish welfare have relied on physical, physiological and/or behavioural measures of biological dysfunction, offering insights into health and functional integrities, yet these approaches face notable limitations. By contrast, affective-based approaches, which use behavioural measures to assess affective state and are widely employed in welfare science, remain underexplored in fishes. Recent advances, such as judgement bias paradigms, provide reliable tools to measure affective states. These support integrative welfare assessments that combine affective evaluations with measures of health and biological functioning. A multi-level approach ensures a comprehensive and robust evaluation of fish welfare, avoiding reliance on any single type of evidence and enabling the exploration of different facets of the welfare construct. While this work synthesizes existing indicators, its main contribution lies in proposing a functional framework that prioritizes affective state evaluation while systematically integrating and validating complementary metrics. By aligning with contemporary ethical and scientific standards, this approach aims to advance the conceptualization and operationalization of fish welfare.

## 1. Why do we need to advance the science of fish welfare?

Fishes inhabit environments markedly different from other vertebrates, facing unique ecological and biological challenges that are often unfamiliar to humans. Consequently, their welfare has historically received less public attention than that of more familiar animals, such as mammals and birds [1]. However, advancing the science of fish welfare is essential to support the growing importance of these organisms in global food systems. Aquaculture represents a potentially sustainable form of food production (e.g. [2–4]), although this potential remains subject to debate and depends on factors such as environmental impact, feed source sustainability and animal welfare considerations [5,6]. Global fish production is projected to substantially increase by 2050, with marine aquaculture expected to contribute a significant share, contingent on technological and sustainability advances [7]. These estimates assume innovations like reduced reliance on fishmeal and fish oil but do not fully account for practical constraints including resource limits, environmental effects and socio-economic challenges that may affect growth feasibility [8,9]. Nonetheless, a considerable expansion of current marine finfish aquaculture production will be necessary to meet future demand [7]. Beyond global food supply, fishes housed in artificial environments play an important role in biodiversity conservation. Zoological institutions implement *ex situ* conservation strategies, including captive breeding programmes, to safeguard species from extinction [10]. These efforts have helped sustain populations of certain taxa like elasmobranchs [11], yet many threatened species still lack viable captive populations [12], highlighting the need to expand such initiatives to support both captive and wild population resilience. Additionally, fishes also represent valuable models in scientific research across diverse disciplines. For example, the zebrafish (*Danio rerio*) has emerged as a key species in biomedical research because of attributes like transparent embryonic development, low maintenance costs and genetic similarities to humans, advancing the development of new clinical tools for diagnosing and treating human diseases [13,14], with a rapidly growing body of scientific literature supporting its use (electronic supplementary material, figure S1). In parallel, the ornamental fish sector is a rapidly growing multibillion-dollar industry [15] dependent on complex supply chains. As demand increases, a major regulatory challenge is the limited capacity to monitor welfare across these networks, which has hindered the development of specific welfare regulations for ornamental fishes [16].

Considering these projections, fish production is expected to grow significantly in the coming years, contributing substantially to global food supply, resource utilization, biodiversity conservation and scientific innovation. However, the welfare of captive animals in increasingly intensive systems raises important ethical concerns [17]. This dynamic is likely to drive public debate and policy discussions, highlighting the need to balance the benefits of intensified fish production with ethical responsibility to ensure the welfare of captive fishes. Ethical concerns in animal welfare are commonly structured around three core principles: (i) expression of natural behaviours, (ii) maintenance of health and biological functioning, and (iii) avoidance of negative states alongside opportunities for positive experiences [17]. The natural-living perspective, for example, focuses on enabling animals to engage in their natural behaviours [18]. However, this approach has inherent limitations, as many natural behaviours are evolutionary adaptations to different environmental pressures, which may include adverse conditions. Recreating environments that elicit such behaviours may inadvertently increase suffering rather than reduce it. As natural behaviours are not always indicative of good welfare, the absence of stereotypies may actually reflect frustration or apathy rather than improved well-being. In some cases, non-stereotyping animals may be more frustrated or apathetic, even unable to cope with captivity. Consequently, this approach has been criticized for inadequately addressing broader ethical concerns about animals’ overall quality of life [19,20] and is rarely used as the sole basis for contemporary welfare assessments. Functioning-based approaches, in contrast, premise that welfare is compromised when biological functions are disrupted [21], relying on physical, physiological and behavioural indicators linked to stress, immune competence and fitness [22]. While these indicators have traditionally been used to infer animal affective states and assess welfare, functioning-based approaches face significant interpretational challenges [23] and require complementary evidence from additional sources [21]. This perspective currently dominates fish welfare science, using various metrics to assess disruptions such as disease, injury, malnutrition and other disturbances to normal biological functioning, all of which are subsequently associated with assessments of fish welfare (e.g. [24–26]). In response to its limitations, a new perspective emerged, offering a promising strategy for assessing animal welfare by directly linking it to an animal’s subjective or affective experiences [27,28]. This affective-based approach has gained prominence in animal welfare science (e.g. [29–31]), operating under the premise that an animal’s welfare may be compromised even if it appears healthy, grows and reproduces, particularly when it experiences subjective suffering.

However, applying these perspectives to modern aquaculture raises important challenges. Fish farming operates at an unprecedented biological scale, with over 200 species, 10 times the diversity of terrestrial livestock, being farmed worldwide [32]. Most remain undomesticated, lacking genetic or behavioural adaptations to captivity [33]. The sector’s rapid expansion and high diversity have widened the gap between the need for species-specific welfare assessment and the capacity of welfare science to meet it, underscoring the challenge of creating tools suited to such extremely high diversity [34]. Thus, developing species-specific frameworks for welfare assessment that integrate physical, physiological, behavioural and affective indicators is essential to generate the knowledge required for tailored strategies, including husbandry protocols, environmental enrichment, optimized feeding and non-invasive health monitoring [35]. In parallel, practical tools must be developed and validated for commercial use, where high densities, complex interactions and economic constraints challenge individual monitoring [36]. Critically, the development of such tools relies on foundational knowledge from species-specific welfare frameworks. Within this context, the ability to reliably infer affective experiences is a key objective in fish welfare science, with cognitive judgement bias emerging as a promising proxy for assessing both affective state and overall welfare [37]. Notably, recent advancements have adapted judgement bias paradigms for fishes [38–40], facilitating the incorporation of the affective-based approach into a comprehensive welfare framework that also includes measures of biological function and health. This integrative model offers a more robust framework of addressing diverse ethical concerns, providing a deeper understanding of fish welfare and advancing the field. Importantly, its application extends beyond captive settings and may also prove valuable for assessing the welfare of wild-caught individuals, which, unlike fully domesticated species, are not genetically selected for or adapted to captivity and may face specific challenges related to capture, handling and transport stress [41]. Additionally, the expansion of marine aquaculture raises concerns about the welfare and ecological integrity of wild populations [42]. Applying a comprehensive welfare framework in these contexts could help identify key risks and guide improvements in management practices.

## 2. Physical indicators of biological dysfunction in fishes

Fish welfare has traditionally been assessed through outcome-based indicators that reflect the biological functioning of the fish. Among these, physical appearance is particularly relevant owing to its relative ease of observation and measurement. The Salmon Welfare Index Model [43] proposes using multiple physical indicators for assessing welfare in Atlantic salmon (*Salmo salar*). This framework includes physical indicators such as emaciation, skin haemorrhages, lesions/wounds, scale loss, eye haemorrhage, exophthalmia, opercular damage, sea lice infection, snout damage, vertebral deformities and jaw deformations. Similar indicators have been applied to other fish species, including gilthead seabream (*Sparus aurata*) [44], lumpfish (*Cyclopterus lumpus*) [45] and rainbow trout (*Oncorhynchus mykiss*) [46]. For elasmobranchs, specific indicators have been identified, including delays or absence in the nictitating membrane reflex, eyeball retraction, spiracle closure, disc contraction, flaring, clasper crossing, stomach or intestinal eversion, and reproductive issues such as premature birth, abortion and dystocia [47]. Despite their usefulness, physical indicators present important limitations. They vary across species and require species-specific identification. Furthermore, accurate scoring often demands experienced assessors, and evaluating all individuals in large-scale systems is difficult, requiring representative sampling. In addition, these indicators usually appear only after welfare has been compromised for an extended period, complicating early detection and intervention.

## 3. Physiological indicators of biological dysfunction in fishes

Outcome-based indicators of fish welfare also incorporate physiological responses driven by the activation of the autonomic and endocrine systems during challenging situations. These responses trigger coordinated physiological changes that help the animal recover and maintain stable internal conditions [48]. Initially, the nervous system releases neuroendocrine hormones, including catecholamines and corticosteroids, which lead to secondary physiological changes such as increased heart and respiration rates and energy mobilization. If the stressor persists, tertiary responses may occur, including the depletion of energy reserves, immune suppression and disruptions in behaviour and reproduction. These prolonged stress responses often manifest as visible changes in the fish’s physical appearance. Classical physiological indicators of fish biological disruption include changes in cortisol and catecholamine levels [49]. For instance, stressed gilthead seabream and European sea bass (*Dicentrarchus labrax*) exhibit elevated plasma cortisol and altered expression of stress-related genes [50,51]. Beyond these, immunity-related biomarkers in dermal mucus serve as stress indicators in gilthead seabream [52]. Intermediary metabolism components encompassing carbohydrates, lipids and amino acids regulation during stress have also been proposed as physiological indicators. For instance, transport-induced stress in gilthead seabream depletes liver glycogen, enhances glycolysis and alters lipid metabolism, shown by changes in triglyceride levels and enzyme activities [53]. Similarly, elevated plasma nonesterified fatty acids have been reported in European eel (*Anguilla anguilla*) and African catfish (*Clarias gariepinus*) following transport [54,55]. As outlined earlier, stress often elicits tertiary responses that impair growth, including the suppression of the growth hormone/insulin-like growth factor system [56] and heightened lipid and amino acid catabolism [57]. Long-term stress is also associated with oxidative stress markers [58] and immune factors in skin mucus [59]. In elasmobranchs, the primary stress response involves the release of catecholamines (adrenaline and noradrenaline) and corticosteroids (1α-hydroxycorticosterone) [60], with secondary responses regulating ion concentrations, pH and urea levels to maintain plasma osmolality [61,62]. However, physiological indicators have notable limitations. They often require specialized equipment and trained personnel, making them costly and less accessible for routine use. Additionally, some analyses are time-intensive, delaying detection and limiting real-time use. Furthermore, certain methods are invasive, potentially exacerbating stress and further compromising fish welfare.

## 4. Behavioural indicators of biological dysfunction in fishes

In addition to physical and physiological changes, behavioural alterations are frequently used to assess disruptions in fish biological functions. For example, stress often reduces feeding motivation and intake, making these parameters valuable indicators documented in various species including European sea bass [63] and zebrafish [64]. Agonistic behaviours associated with dominance hierarchies are also used as indicators of biological disruption, particularly when they lead to injuries or fin damage [65]. Swimming behaviour often reflects how fishes perceive and respond to their environment, although its relevance varies with species and context. For instance, hypoxia decreases swimming speeds in white sturgeon (*Acipenser transmontanus*) [66] and Atlantic cod (*Gadus morhua*) [67], whereas brook charr (*Salvelinus fontinalis*) increase speed under low oxygen conditions [68]. Stereotypic behaviours, defined as repetitive and invariant actions with no apparent function, are also considered relevant indicators of biological dysfunction. Although evidence of stereotypy in fish is limited, examples include circular or triangular patterns of compulsive swimming in African catfish [69], vertical swimming loops in Atlantic halibut (*Hippoglossus hippoglossus*) [70] and vacuum pit digging in Mozambique tilapia (*Oreochromis mossambicus*) [71]. Conversely, consistent expression of species-typical behaviours like environmental exploration [72] or jumping and play-like behaviours [73] may indicate adequate biological functioning. However, these natural behaviours are context- and species-dependent, and their presence does not necessarily indicate good welfare, just as the absence of stereotypies does not guarantee a better condition. Still, regular occurrence of natural behaviours under suitable conditions suggests that basic behavioural needs are met. In elasmobranchs, behavioural indicators of biological dysfunction include abnormal swimming patterns: rapid, constant swimming; erratic, jerky movements; slow, laboured swimming with exaggerated head motions; poor navigation; vertical swimming (sometimes breaking the water surface) and tight circular swimming or ‘looping’ [74]. Behavioural indicators are easily observed and well-suited for onsite assessment but present significant limitations, including temporal and group variability, difficulty in quantification and the need for skilled observers. Some behaviours may function as adaptive coping mechanisms that promote welfare, while others may be maladaptive and detrimental to welfare. The distinction between adaptive (normal) and abnormal behaviours is often ambiguous.

## 5. Specific indicators of affective states in fishes

**Figure 1.**
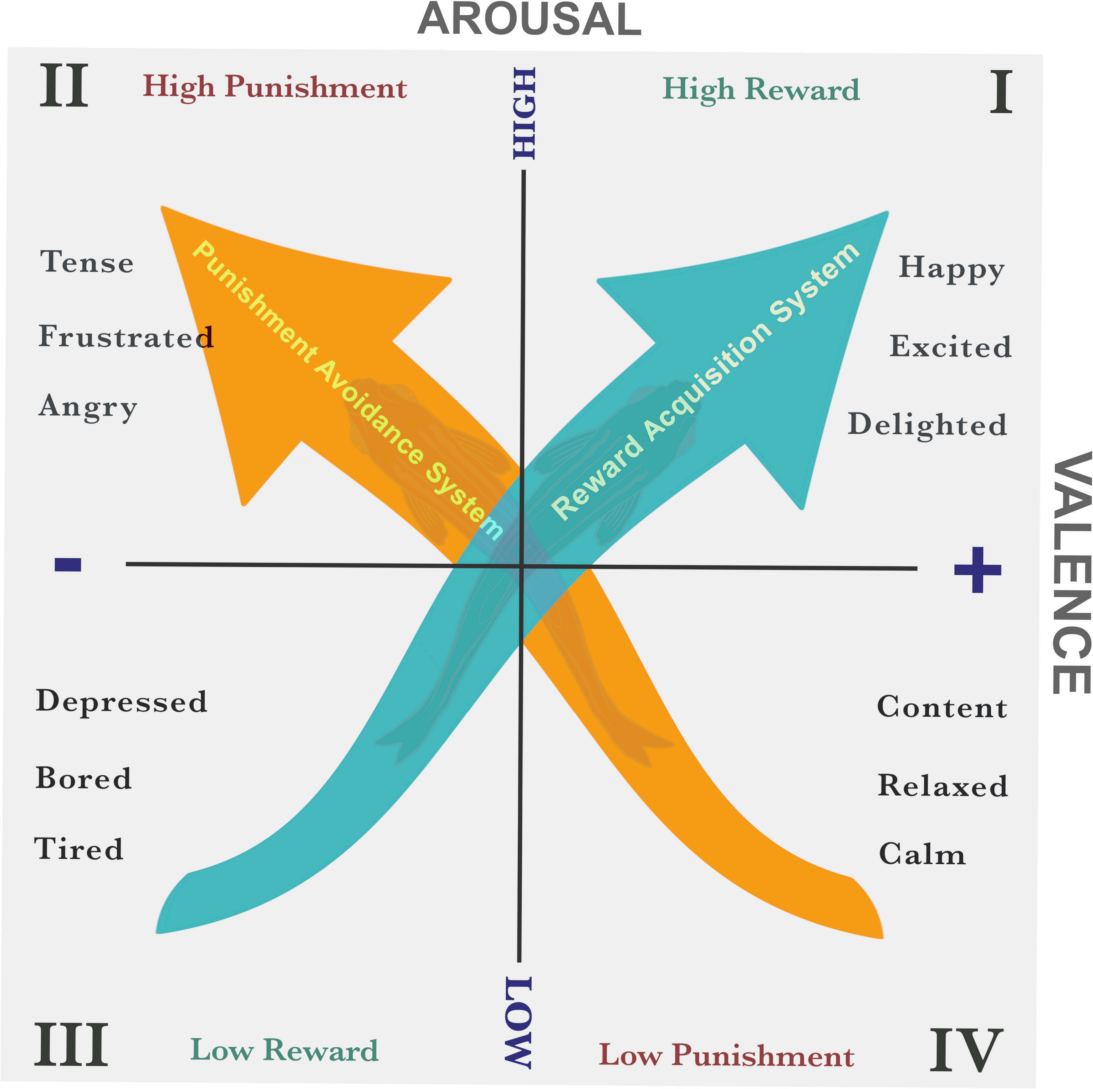
Core affect dimensional model of emotion, adapted from Mendl *et al*. [30]. The putative reward acquisition and punishment avoidance systems are superimposed onto the dimensional core affect model. Four fundamental core affect states (high-reward, low-reward, high-punishment and low-punishment) are predicted by an operational definition of animal affect and correspond to the quadrants of the core affect space. These states are associated with the activation (or low activation threshold) or deactivation (or high activation threshold) of reward acquisition and punishment avoidance systems, whose activity aligns at a 45° angle to the core affect axes. The direction of the arrows represents changes in affective state. Maximizing reward system engagement and minimizing punishment system activation promotes positive valence and improves welfare.

In addition to traditional physical, physiological and behavioural indicators of biological functioning, certain behavioural metrics observed in structured contexts (e.g. behavioural tests or paradigms) have been proposed as indicators of affective states, as they provide insight into the emotional valence of an animal’s experience. Similar to animal welfare, the concept of animal affect has long generated debate around conceptualization and terminology. Over the years, several attempts to operationally define affective states in animals have been proposed, primarily using reinforcement learning-based approaches (e.g. [75]). More recently, Rolls [76] introduced an operational definition of animal affect, later refined by Mendle & Paul [77], which identifies valence as the core characteristic of affective states. According to these authors, affective states are elicited by rewards, punishers or their predictive cues. In this context, rewards are stimuli that animals actively seek or work to obtain, while punishers are those they strive to avoid. From this definition, four fundamental affective states can be identified: (i) positive states arising from a reward or its predictors, (ii) positive states from the termination or omission of an anticipated punishment, (iii) negative states triggered by a punishment or its predictors, and (iv) negative states from the termination or omission of an anticipated reward. These states map closely with locations in several dimensional models of human affect, where emotions are explained by the interplay of underlying dimensions or neurobehavioural systems. For instance, the dimensional model of core affect [78,79] explains human emotions through two key dimensions: valence, which ranges from positive to negative and indicates whether an experience is pleasant or aversive; and arousal, which reflects the level of activation, from low (calm, relaxed) to high (excited, stressed). Extended models suggest these dimensions correspond to neurobehavioural systems associated with reward acquisition and punishment avoidance (e.g. [80]), which are conceptualized as lying at a 45° angle to the core affect axes, illustrating their interaction with valence and arousal ([80,81]; figure 1).

Integrating the four affective states model for animals with dimensional models of human emotion offers a scientifically grounded approach for studying animal affect. This combined approach allows researchers to empirically determine an animal’s location in core affect space by assessing activity levels within neurobehavioural systems, which enables a systematic mapping of affective states. For instance, exposure to a reward or its predictors elevates reward acquisition activity, producing a heightened reward state, whereas the omission or loss of an expected reward decreases this activity, resulting in a diminished state. Similarly, punishment or its predictors increase punishment avoidance activity, leading to a heightened punishment state, while successfully avoiding or omitting an anticipated punishment reduces this activity, generating a state of diminished punishment. Direct activation of either system (reward acquisition or punishment avoidance) generally results in heightened arousal, preparing the organism for immediate, vigorous action to secure the reward or evade the punisher [80]. By contrast, lower arousal states occur when these systems are indirectly activated, as in omission or avoidance. Additionally, valence is associated with whether each system is active or inactive: increased reward acquisition activity or decreased punishment avoidance activity generates positive valence, while heightened punishment avoidance or reduced reward acquisition activity results in negative valence [80]. Shifts in system activity correspond to distinct, transient core affect responses.

Building on the hypothesis that animals, like humans, experience central affective states, it is reasonable to assume these states can be experimentally inferred. In psychological research on human emotion, subjective experiences are typically assessed through verbal reports. However, given animals’ inability to communicate verbally, developing reliable proxy measures to infer their affective states has become an important goal in animal welfare science. Emotions are recognized as multifaceted, involving not only subjective experiences but also behavioural and physiological changes (e.g. [82]). For instance, fear involves not only the subjective feeling of fear but also freezing or fleeing behaviours and physiological changes such as fluctuations in heart rate, blood pressure and glucocorticoid levels. Based on this, physiological and behavioural measures have traditionally been used to assess animal affect, including in fishes. Yet these indicators present important interpretational limitations [23]. For instance, some measures effectively capture arousal but fail to differentiate between positive and negative states. An elevated heart rate, for example, could indicate excitement in anticipation of a reward or fear when facing a potential punishment. As discussed previously, emotional valence, a key dimension of the core affect model, reflects the active or inactive state of either reward acquisition activity or punishment avoidance systems, making it essential for accurately assessing affective states in animals. In this context, because a single measure can reflect multiple affective states, it also becomes difficult to predict in advance how it should vary with affect, complicating the formulation of *a priori* hypotheses. For instance, in open field tests, increased activity could suggest either exploratory behaviour (positive valence) or escape-oriented responses (negative valence), making interpretation and cross-species comparisons more challenging. Further challenges arise from discrepancies between self-reported emotions and physiological indicators. For example, some individuals display clear physiological signs of an emotional response despite reporting no change in their subjective experience, while others report strong emotions without corresponding physiological changes [83,84]. These inconsistencies question how reliably physiological and behavioural indicators capture subjective states. Moreover, physiological and behavioural indicators often provide limited insight into positive affect, a growing focus in animal welfare. This gap further complicates efforts to comprehensively evaluate an animal’s overall welfare.

These limitations have prompted the integration of cognition as the fourth component of emotion alongside subjective, behavioural and physiological aspects. Research in human psychology shows that cognitive processes such as attention, learning, memory and decision-making both shape and are shaped by emotional states. One such process is cognitive appraisal, which involves the evaluation of stimulus features (e.g. intrinsic valence, novelty, prediction error and control) and modulates the emotional responses displayed [85]. In animal research, this approach is applied by exposing individuals to specific cues signalling stimulus checks (e.g. suddenness, unfamiliarity, unpredictability or unpleasantness) that correspond with human-reported emotional states (e.g. fear). Assuming these appraisals are similarly linked to emotions across species, this framework offers an *a priori* approach for identifying affective states in animals by linking their behavioural and physiological responses to these specific cues with predicted affective states (e.g. [86,87]). Empirical evidence supports the occurrence of all stimulus evaluation checks in fishes. For instance, intrinsic valence has been demonstrated through learnt approach/avoidance behaviours in gilthead sea bream and European sea bass [88,89]. Additionally, fishes have shown sensitivity to novelty-related cues: (i) predictability, which modulates behavioural and physiological responses to aversive and appetitive stimuli in Mozambique tilapia [90]; (ii) familiarity with conspecifics, influencing exploratory behaviour and responses to territorial intrusion also in tilapia [91]; and (iii) controllability, demonstrated in rainbow trout, where individuals that avoided social defeat had lower cortisol levels [92]. Finally, prediction error (i.e. the discrepancy between expected and actual outcomes) has been documented in rainbow trout and Atlantic salmon using a reward omission paradigm [93,94], reflecting the capacity to update expectations based on new information, a key component of adaptive stimulus evaluation.

Beyond the role of cognitive processes in shaping emotions, emotional states can also influence cognition. Research in human psychology has consistently reported that emotional valence influences key cognitive functions such as attention, memory and judgement. For instance, individuals in negatively valenced states (e.g. anxiety) display heightened attention to threatening stimuli [95]. Similarly, affective states influence memory retrieval, with positive emotions favouring the recall of positive memories, while sadness or depression promote the recall of negative ones [96]. Negative affective states also lead to pessimistic judgements about future events or ambiguous stimuli, whereas positive states encourage optimism [97]. This relationship between affective state and decision-making under ambiguity has attracted significant attention in animal research, and judgement bias paradigms have been developed for a variety of species (e.g. [98–101]), consistently serving as reliable indicators of affective states (e.g. [32,99,100]). In animals, judgement bias is conceptualized as a decision-making process in which some individuals interpret ambiguous stimuli positively (optimism), while others interpret them negatively (pessimism). This process is typically assessed using judgement bias tasks, where subjects respond to ambiguous cues positioned between two anchor cues that differ in the valence of their associated outcomes (e.g. positive versus negative or positive versus neutral). Responses to ambiguous probes indicate the extent to which an animal perceives the ambiguous cue as either positive or negative. This approach offers several advantages over traditional indicators of affective states as it allows for precise measurement of emotional valence (positive or negative), provides *a priori* predictions (based on human studies) about the co-variation of cognitive performance and emotion and is applicable across species, while also enabling the assessment of positive affective states. In fishes, this paradigm has been successfully applied to zebrafish, where generalization responses across cues have validated its use in this species [39,102]. Stimulus generalization plays a key role in the interpretation of ambiguous stimuli in judgement bias paradigms, indicating that zebrafish can discriminate between stimuli predicting positive and negative outcomes. Studies using this assay show that judgement bias in zebrafish is a consistent behavioural trait over time [103]. Further research has revealed associations between this trait and factors related to fitness [104] and disease susceptibility [103], as well as a causal relationship between telomere attrition and pessimism [39]. Moreover, the assay demonstrated a direct interaction between affective states and decision-making under ambiguity in both zebrafish [103] and European sea bass [40], highlighting the potential of judgement bias tests to advance understanding of fish welfare. Although not yet developed for elasmobranchs, their cognitive abilities [105] suggest judgement paradigms could be adapted for this taxon.

Complementing cognitive processes, such as appraisal and judgement bias, preferences for specific environmental conditions or resources and the willingness to work to obtain them, have also been proposed as indicators of the cognitive dimension of emotion. Marian Dawkins emphasized using behavioural indicators of animal preferences to assess *what they want*, arguing that their choices offer a pragmatic way to infer affective experiences and welfare [106,107]. However, Ian Duncan argued that identifying preferences alone may be insufficient, and that measuring motivation strength (i.e. how much effort an animal invests to obtain or avoid a stimulus) is also essential to assess affective states and set welfare priorities [108]. These ideas underpin the development of preference and motivation tests, widely used to infer *what animals want*, including fishes. Within preference testing in fishes, one example is the preference index (PI), originally developed for Mozambique tilapia, which uses a history-based method integrating repeated choices over time and giving more weight to recent decisions to estimate both valence and preference strength [109]. Although applied across species and contexts [110], the PI’s direct use in commercial operations remains limited, positioning it primarily as an experimental tool for developing future industry welfare protocols. Such tests are sensitive to factors like perceptual salience, prior experience and transient internal states, which may obscure the difference between stable preferences and short-term choices. Moreover, demonstrated preferences do not always reflect a welfare benefit or positive affect, as animals may choose suboptimal or even harmful options depending on context. Nevertheless, when carefully designed and interpreted, these tests remain valuable tools for inferring the cognitive dimension of affect and informing welfare assessment.

## 6. How can we comprehensively assess welfare in fishes?

The subjective or affective perspective on welfare has become the dominant framework in animal welfare science (e.g. [29–31]), highlighting the importance of understanding and assessing emotional processes in animals. Emerging measures of affective state, such as judgement bias, have provided reliable welfare indicators and have been effectively applied to fish species (e.g. [39,40]). However, there is growing emphasis on validating these indicators to ensure they accurately reflect welfare state. Tasks assessing preference and ‘willingness to work’ for specific resources (i.e. motivation), which align with reinforcement-based definitions of affect, have become central to this validation process [111,112]. When interpreted in the context of the animal’s evolutionary heritage, these measures show convergent validity with other welfare indicators (e.g. [112–114]), establishing a gold standard for interpreting welfare data. Validation assumes that animals in artificial environments can integrate relevant inputs and make decisions that reflect their own best interests, positioning preferences and motivation as particularly meaningful welfare measures that should correlate with other valid welfare indicators. Building on these principles, we propose a functional framework for assessing fish welfare that prioritizes affective state evaluation through validated indicators. The primary objective is to determine whether decision-making under ambiguity and preference/motivation for resources converge to provide corroborative evidence of welfare. A positive relationship between these measures would suggest that access to preferred environments or resources enhances affective state, leading to a more optimistic judgement bias. This would support the validity of judgement bias as a welfare indicator and show that welfare improves through interaction with preferred resources. Supporting this, recent studies showed that farmed chickens (*Gallus gallus domesticus*) housed in preferred conditions, pens enriched with specific features and positive stimuli, displayed a more optimistic judgement bias than those in non-preferred environments [114]. Interestingly, this association emerged after short-term exposure but diminished over time, suggesting hedonic adaptation. This finding highlights the need to include both short- and long-term assessments in longitudinal study designs when evaluating potential welfare indicators.

Animal welfare is a complex and multifaceted phenomenon that requires a comprehensive approach to assessment. Since no single measure can fully capture the nuances of an animal’s welfare, a multilevel analysis is essential. Accordingly, the second aim of the proposed approach is to determine whether additional indicators of biological dysfunction, such as physical, physiological and behavioural measures, are significantly associated with individual measures of preference and motivation in fishes. Similar studies have evaluated the validity of over 100 welfare indicators to ensure a comprehensive assessment [111,114]. As previously discussed, validating indicators through animals’ preferences or ‘willingness to work’ assumes they can associate specific environments or resources with welfare, identifying which indicators best reflect what animals find rewarding. In other words, since preference and motivation are intrinsically linked to the affective dimension of welfare, validated physical, physiological and behavioural indicators can indirectly reflect affective state and provide insight into overall health. These levels of analysis are exemplified in mammals, such as fur-farmed minks (*Mustela vison*), where restricting access to a highly preferred resource (a swimming pool) led to increased urinary cortisol levels, reflecting frustration owing to the inability to swim [113]. Similarly, farmed chickens preferred environments where they displayed calmer behaviours during novel object tests (e.g. perching, pecking, walking and tonic immobility) [111]. These established fear indicators in chickens [115] suggest preferred environments reduced fear responses. Notably, the same study found that corticosterone, despite being widely used as welfare indicator in laying hens, did not predict environmental preferences, reinforcing scepticism about its value in the welfare of this species (e.g. [116]). These findings highlight the importance of validating welfare parameters by examining their relevance to animals’ individual preferences and motivation.

**Figure 2.**
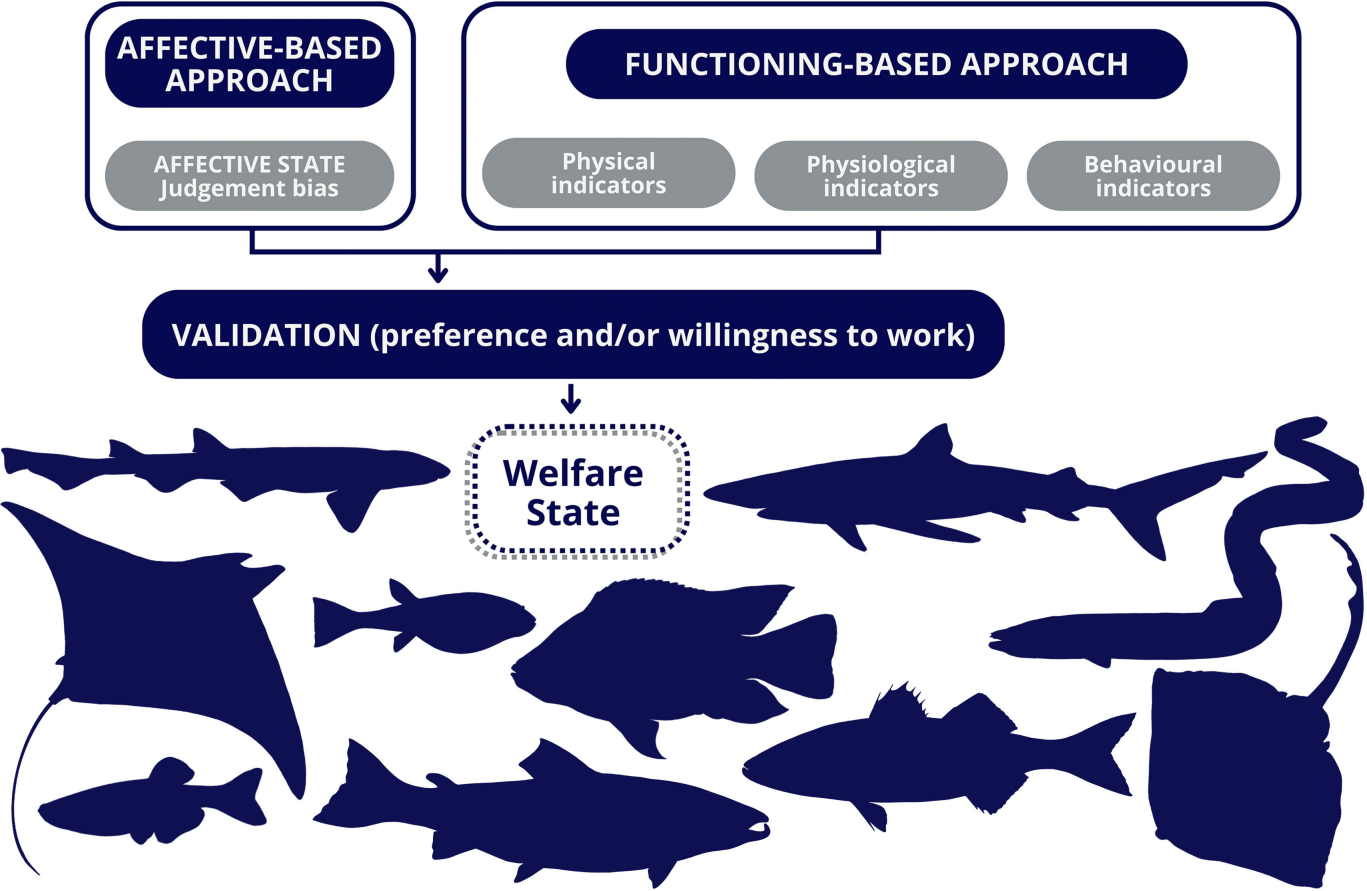
Schematic representation of the proposed framework for fish welfare assessment, which integrates animals’ responses to different environments or procedures with decision-making that reflects their own best interests. This approach prioritizes the evaluation of the fish’s affective state as a central component of welfare assessment, alongside traditional physical, physiological and behavioural indicators. Behavioural indicators are considered in both domains: while some reflect biological functioning (e.g. abnormal or suppressed behaviour), others, such as those measured in judgement bias tasks, are used to infer affective states. Welfare measures are further validated through fish preferences or their ‘willingness to work’ for specific resources or environments.

The introduction of bubble curtains as environmental enrichment, believed to benefit welfare in certain fish species [117], offers an excellent opportunity to illustrate the proposed framework for welfare assessment in fishes (figure 2). This example integrates a validated affective evaluation of the fish with physical, physiological and behavioural measures, each further validated through preference and motivational tasks. Notably, the importance of considering fish preferences when designing enrichment strategies was highlighted in earlier studies [109]. A potential experimental design could involve sequential exposure to two phases over a 15 week period, with an optional third phase focusing on long-term exposure to the experimental conditions. In phase 1, lasting 10 weeks, group-housed fish are kept in identical conditions with access to two environments: one enriched with a bubble curtain and the other a control environment without bubbles. These environments are situated in parallel tanks connected by a tunnel, allowing the fish to move freely between them. This phase aims to minimize novelty effects and allow stable preferences to emerge through repeated voluntary exploration. Although preferences may fluctuate owing to habituation or changes in internal state, such variation is part of the natural process through which consistent affective evaluations emerge. Importantly, when one environment is clearly more rewarding or less aversive, transient shifts in preference are unlikely to result in long-term selection of the less favourable option, enhancing the reliability of subsequent assessments. Phase 2 involves a short-term, five week exposure to fixed housing conditions. Fish are randomly allocated to either the bubble-enriched or the control environment. Welfare data collection occurs in the final week, including daily choice tests with access to both environments for seven days. Preference will be quantified using the PI, a history-based metric that gives greater weight to recent trials while incorporating the full behavioural record [109]. Positive PI values indicate preference, while negative values indicate non-preference. This approach minimizes the influence of isolated or spurious choices and yields a robust estimate of stable individual preferences over time. A preference for the bubble-enriched tank would indicate that fish find this environment more rewarding. Complementarily, motivation could be assessed through requiring effort to access the enriched environment. For example, fish could be required to push a door [118], swimming against a current [119], risk predator exposure [120], interact with a non-opening door [121] or overcome psychologically aversive conditions [121]. A high willingness to overcome such challenges would indicate that fish value the bubbles, reflecting their motivational state. As discussed earlier, the correlation between judgement bias and preference or motivation is crucial for validating this welfare indicator. For instance, if the bubble curtain induces a positive affective state, fish housed in the bubble-enriched environment should display a more optimistic judgement bias, aligned with their preference for this environment. A correlation between these measures would strengthen the interpretation that preference for the enriched environment reflects affective experience, supporting judgement bias as a valid welfare indicator. In addition, physical, physiological and behavioural indicators of biological dysfunction will be collected to ensure a comprehensive welfare assessment. For instance, physiological measures such as cortisol levels or heart rate will provide insight into stress, while behavioural observations like activity, aggression and stereotypies, and physical signs, such as lesions, wounds and scale loss, will inform on overall health. These indicators should also correlate with the fish’s preferences or ‘willingness to work’ for access to the enriched environment, further validating the selected welfare measures.

## 7. Limitations of the approach

Despite its conceptual strengths and its value for generating foundational knowledge to inform welfare assessment in fishes, this functional approach faces practical limitations that constrain its application in large-scale settings. A key challenge lies in judgement bias tasks, which offer robust insights into affective states but require time-intensive, complex training procedures. In practice, a substantial proportion of fish may fail to learn the discrimination task, often leading to small sample sizes. These demands limit feasibility in commercial contexts. Nevertheless, given the valuable information it yields, such behavioural paradigms have been proposed for welfare assessment in aquariums and zoos, where individualized monitoring is more feasible [122]. With appropriate protocol adaptations and institutional support, this methodology holds strong potential in such settings. To enhance scalability, alternative or complementary indicators may offer more practical proxies of affective state. For example, startle response paradigms from birds [123] could inspire analogous fish assays but require rigorous validation to account for major inter-taxa differences. Integrating such refined protocols and/or complementary indicators into the proposed welfare framework could facilitate broader implementation and routine aquaculture monitoring.

Another challenge is the reliance on invasive procedures, such as blood sampling for physiological analyses and behavioural tests involving mild punishment. Although widely used and scientifically robust, these methods raise ethical concerns and pose logistical difficulties owing to technical complexity and resource demands. Nevertheless, these approaches yield valuable information: blood analyses provide reliable indicators of stress, and reward- or punishment-based tests remain among the few validated methods for assessing emotional valence. To enhance feasibility and ethical acceptability, future research should prioritize the refinement and validation of less invasive alternatives. Promising options include hormone measurements in skin mucus [124] and complementary indicators of affective states such as the startle response [123], though further validation across species and contexts is still needed.

Beyond methodological challenges, structural factors in aquaculture further complicate welfare assessment. The sector’s rapid growth often surpasses scientific understanding, especially at the species level, making it particularly difficult to scale welfare approaches across the diverse range of farmed fishes and production systems. Unlike terrestrial farming, which focuses on a few well-domesticated species, aquaculture involves hundreds [32], many of which have only recently been introduced to captivity and whose specific needs remain poorly understood [33]. This lack of species-specific data, combined with the high diversity of farmed fishes, challenges the development of standardized welfare tools and increases the risk of inadequate welfare protection across species [125,126]. However, affective indicators can help identify environmental conditions or resources that fishes perceive as rewarding or aversive, offering a scientific foundation for more species-specific welfare practices. While such tools may be too resource- and time-intensive for routine use in commercial farms, they remain essential for addressing the challenges posed by the high diversity of farmed fish species and can also provide a foundation for developing simplified, cost-effective proxies.

## 8. Conclusions

In animal welfare science, traditional methods often evaluate animals’ responses to various environments or procedures. By contrast, an alternative perspective focuses on the importance of animal decision-making, based on the assumption that even domestic animals in artificial settings can integrate various inputs and make choices that reflect their own best interests. Building on these two perspectives, we propose a novel framework for fish welfare assessment that combines both perspectives. Our approach prioritizes the evaluation of the fish’s affective state as a core component of welfare assessment, integrating it with traditional physical, physiological and behavioural indicators of biological dysfunction. Crucially, these welfare measures gain relevance only when validated by fish preferences or their ‘willingness to work’ for specific resources or environments, thereby linking them directly to the affective dimension. This multi-level analysis ensures a comprehensive and reliable evaluation of fish welfare, capturing emotional valence beyond what conventional indicators reveal and providing a more precise and insightful assessment. Future research should explore both alternative or complementary methods for assessing judgement bias and refined training protocols to increase sample sizes, along with the development and validation of non-invasive indicators. Furthermore, the use of multiple-choice paradigms instead of binary tests could offer greater decision-making freedom and help reveal more nuanced, consistent preferences. In this context, it is important to avoid relying on a single or a limited number of tests, as consistent preference patterns may take time to emerge [109]. Additionally, expanding the range of welfare indicators and exploring their inter-relationships could help identify distinct facets of fish welfare. Further validation across species is essential for developing a more comprehensive understanding of the welfare construct. This is especially relevant as welfare may not correspond to a single, unified construct defined by one coherent set of correlated indicators. Instead, it may be better represented as multiple distinct facets, each reflecting separate dimensions of welfare rather than a single global measure.

## Data Availability

Supplementary material is available online [127].
